# Supplementary material for: HIVprotI: an integrated web based platform for prediction and design of HIV proteins inhibitors
Source: J Cheminform. 2018 Mar 9;10:12. doi: 10.1186/s13321-018-0266-y (PMC5845081; doi:10.1186/s13321-018-0266-y)
Supplement: Supplementary file 2 — Additional file 2. Source code of HIVProtI web server. [file 13321_2018_266_MOESM2_ESM.zip › HIVprotI_Source-code/hivprotI/weka-3-6-6/doc/weka/estimators/package-summary.html]

weka.estimators


---


|  |  |  |
| --- | --- | --- |
| **Overview** \| **Package** \| Class \| **Tree** \| **Deprecated** \| **Index** \| **Help** \| **Weka's home** | |  |
| **PREV PACKAGE**   **NEXT PACKAGE** | **FRAMES**    **NO FRAMES**     **All Classes** |  |


---

## Package weka.estimators

| **Interface Summary** | |
| --- | --- |
| **ConditionalEstimator** | Interface for conditional probability estimators. |
| **IncrementalEstimator** | Interface for an incremental probability estimators. |

| **Class Summary** | |
| --- | --- |
| **CheckEstimator** | Class for examining the capabilities and finding problems with estimators. |
| **CheckEstimator.AttrTypes** | class that contains info about the attribute types the estimator can estimate estimator work on one attribute only |
| **CheckEstimator.EstTypes** | public class that contains info about the chosen attribute type estimator work on one attribute only |
| **DDConditionalEstimator** | Conditional probability estimator for a discrete domain conditional upon a discrete domain. |
| **DiscreteEstimator** | Simple symbolic probability estimator based on symbol counts. |
| **DKConditionalEstimator** | Conditional probability estimator for a discrete domain conditional upon a numeric domain. |
| **DNConditionalEstimator** | Conditional probability estimator for a discrete domain conditional upon a numeric domain. |
| **Estimator** | Abstract class for all estimators. |
| **EstimatorUtils** | Contains static utility functions for Estimators. |
| **KDConditionalEstimator** | Conditional probability estimator for a numeric domain conditional upon a discrete domain (utilises separate kernel estimators for each discrete conditioning value). |
| **KernelEstimator** | Simple kernel density estimator. |
| **KKConditionalEstimator** | Conditional probability estimator for a numeric domain conditional upon a numeric domain. |
| **MahalanobisEstimator** | Simple probability estimator that places a single normal distribution over the observed values. |
| **NDConditionalEstimator** | Conditional probability estimator for a numeric domain conditional upon a discrete domain (utilises separate normal estimators for each discrete conditioning value). |
| **NNConditionalEstimator** | Conditional probability estimator for a numeric domain conditional upon a numeric domain (using Mahalanobis distance). |
| **NormalEstimator** | Simple probability estimator that places a single normal distribution over the observed values. |
| **PoissonEstimator** | Simple probability estimator that places a single Poisson distribution over the observed values. |

---


|  |  |  |
| --- | --- | --- |
| **Overview** \| **Package** \| Class \| **Tree** \| **Deprecated** \| **Index** \| **Help** \| **Weka's home** | |  |
| **PREV PACKAGE**   **NEXT PACKAGE** | **FRAMES**    **NO FRAMES**     **All Classes** |  |


---
